# Strategic decision-making for redo cardiac surgical patients with biventricular heart failure – description of a surgical technique

**DOI:** 10.1051/ject/2025059

**Published:** 2026-06-19

**Authors:** Svetlana Novikova, Shyryn Kenzhebekova, Maksat Faizulla, Abdurashid Mussayev, Nail Khissamutdinov, Zhuldyz Nurmykhametova, Yuriy Pya

**Affiliations:** 1 Department of Adult Cardiac Surgery, UMC Heart Center Astana 010000 Kazakhstan; 2 Department of Interventional Cardiology, UMC Heart Center Astana 010000 Kazakhstan; 3 Department of Adult Cardiology, UMC Heart Center Astana 010000 Kazakhstan; 4 Department of OR with Mechanical Circulatory Support, UMC Heart Center Astana 010000 Kazakhstan

**Keywords:** Transcatheter Aortic Valve Replacement, Balloon expandable transcatheter heart valve, Mechanical Aortic Valve, Left Ventricular Assist Device, Heart Mate 3

## Abstract

*Background*: Left ventricular assist devices (LVADs) play a critical role in managing end-stage heart failure (HF), serving as a bridge to transplantation (BTT) or as destination therapy (DT). Patients with prior mechanical valve replacements who later require LVAD support present unique surgical challenges, including increased thrombotic risk, hemodynamic mismatch, and prolonged operative time. To address these concerns, direct balloonexpandable transcatheter heart valve (B/Ex-THV) implantation offers a minimally invasive alternative for high-risk redo cases. *Objective*: To evaluate the feasibility, safety, and hemodynamic performance of direct B/Ex-THV implantation combined with HeartMate 3 LVAD in high-risk patients with prior mechanical valve surgery or native aortic valve disease. *Methods*: This retrospective case series included 16 patients undergoing concomitant HeartMate 3 LVAD and direct B/Ex-THV implantation between April 2022 and October 2023. Nine patients received THVs anchored within prior mechanical prostheses (valve-in-frame), while seven underwent implantation into the native aortic annulus (valve-in-ring). Procedural metrics, hemodynamic outcomes, complications, and survival were analyzed. *Results*: All procedures were successfully completed. The valve-in-frame group demonstrated stable mid-term hemodynamics with low mean gradients (5.0 ± 1.3mmHg) and preserved effective orifice area. No significant paravalvular leak or structural deterioration was observed. At 30 days, 93.75% of patients survived; 12 and 24-month survival rates were both 75%. No valve migration, thrombosis, or hemolysis occurred. One patient who underwent valve-in-frame implantation subsequently underwent successful orthotopic heart transplantation 12 months later. *Conclusion*: Direct B/Ex-THV implantation with concurrent LVAD surgery is a feasible and safe strategy in complex redo patients. It minimizes ischemic time, preserves hemodynamic function, and aligns with ISHLT recommendations, offering a promising approach for managing advanced HF in patients with previous valve interventions.

## Introduction

Left ventricular assist devices (LVADs) have emerged as a cornerstone in the management of end-stage heart failure (HF), providing circulatory support either as a bridge to heart transplantation (HT) or as destination therapy (DT) for patients ineligible for transplant. While HT remains the gold standard for advanced HF, its widespread application is constrained by the limited availability of donor organs and patient-specific contraindications [1, 2]. As emphasized in the latest 2023 International Society for Heart and Lung Transplantation (ISHLT) guidelines, LVAD therapy has become increasingly integral to long-term HF management, significantly improving survival and quality of life in selected patients [3]. In recent years, an increasing number of patients with prior mechanical mitral or aortic valve implantation have required LVAD support due to progressive HF [4]. Managing these patients poses significant challenges, particularly due to the risks associated with repeated valve replacement procedures, including prosthetic valve dysfunction, thrombosis, and structural deterioration [5]. Thus, transcatheter heart valve (THV) therapy has gained more attention as a less invasive alternative for high-risk surgical candidates. According to ISHLT guidelines, THV implantation is now recognized as a viable option in patients with pre-existing valvular prostheses undergoing LVAD placement, provided that anatomical and hemodynamic considerations are favorable [3, 6]. According to the 2023 International Society for Heart and Lung Transplantation (ISHLT) guidelines, mechanical prosthetic valves are not recommended in patients undergoing left ventricular assist device (LVAD) implantation due to the high risk of thrombosis, valve dysfunction, and increased anticoagulation burden. In patients with prior mechanical valve replacement, managing concurrent valvular and LVAD implantation presents significant challenges, including: (1) high risk of valve thrombosis due to altered hemodynamics in LVAD-supported circulation; (2) increased bleeding risks with systemic anticoagulation, necessary for mechanical prostheses; (3) hemodynamic compromise caused by prosthetic valve stenosis or dysfunction, especially in cases of bioprosthetic degeneration; and (4) prolonged surgical and cardiopulmonary bypass (CPB) time, which negatively impacts right ventricular (RV) function postoperatively [3]. Given these considerations, bioprosthetic and transcatheter valve options are preferred in patients requiring LVAD therapy [3, 4, 7]. The direct balloon-expandable transcatheter heart valve (B/Ex-THV) approach offers a minimally invasive alternative to conventional valve replacement in redo cases with significant comorbidities [4]. To address these complexities, novel approaches such as direct implantation of B/Ex-THV have been explored. The MyVal (Meril) B/Ex-THV has demonstrated potential in reducing perioperative risks associated with multiple re-replacements by minimizing aortic cross-clamp time, reducing blood loss, and shortening ICU stay [8]. Advances in THV technology, including improvements in valve design, delivery systems, and procedural techniques, have further enhanced long-term outcomes and procedural safety, reinforcing its role in contemporary HF management [9, 10]. This evolving paradigm – integrating LVAD and THV therapy – offers a new hope for patients with complex HF and valvular disease. The growing adoption of these interventions reflects a shift toward individualized, multidisciplinary management strategies aimed at optimizing survival and quality of life [5, 11].

In this case series, we evaluated the feasibility and safety of concomitant HeartMate 3 LVAD implantation and direct balloon-expandable transcatheter heart valve (B/Ex-THV) deployment in sixteen high-risk, end-stage heart failure patients. Nine of them with B/Ex-THV anchored within the frame of a pre-existing mechanical prosthesis (AV/MV) and the rest performed into the native aortic valve annulus. By performing both interventions in a single operative field, we aimed to minimize total myocardial ischemic time and reduce postoperative morbidity. Our prespecified objectives were to:

1. Assess procedural feasibility and safety, including success of leaflet fracturing, frame preparation, and THV anchoring without embolic or structural complications.
2. Quantify immediate and mid-term hemodynamic performance, as measured by transvalvular gradients, effective orifice area, and absence of paravalvular leak.
3. Detail key technical considerations, encompassing preoperative CT–based frame sizing, valve selection strategy, and deployment technique under imaging guidance.

## Material and methods

We conducted a retrospective analysis of patients with end-stage heart failure who underwent combined direct balloon-expandable transcatheter heart valve (B/Ex-THV) implantation and HeartMate 3 LVAD implantation at our center. All patients treated between April 2022 and October 2023 (*n* = 15) were included following approval from the Institutional Review Board (IRB No. 4/2022, dated April 20, 2022), and written informed consent was obtained from all participants. Among these, 10 patients had a history of prior open-heart surgery, 8 were on a bridge-to-transplantation (BTT) strategy, and 7 were treated as destination therapy (DT) candidates. Procedural outcomes and survival rates were analyzed.

The choice between valve-in-frame and valve-in-ring implantation was based on preoperative anatomical and clinical assessment. Valve-in-frame implantation was selected in redo patients with a prior mechanical prosthesis, where the annular geometry and prosthetic sewing ring provided a stable anchoring zone. Furthermore, in redo patients, removal of a previously implanted prosthetic frame would have markedly prolonged operative and cardiopulmonary bypass time, potentially compromising right ventricular function; therefore, valve-in-frame implantation was favored in such cases. Valve-in-ring implantation into the native aortic annulus was performed when the annulus was preserved, free from severe calcification, and allowed safe expansion of the balloon-expandable THV.

Clinical factors, such as previous surgical history, hemodynamic stability, and the anticipated risk of paravalvular leakage, were also considered in the decision-making process.

Patient selection was conducted by a multidisciplinary heart team – including cardiac surgeons, interventional cardiologists and radiologists – based on preoperative CT, echocardiographic assessment, and overall surgical risk profile. This structured approach ensured that the choice of technique reflected the patient’s individual anatomy and clinical condition rather than operator preference, aiming to minimize perioperative risk and optimize long-term hemodynamic performance.

Two B/Ex-THV implantation techniques have been described:

1. Direct B/Ex-THV implantation into the mechanical prosthesis frame (*valve-in-frame*) – 9 cases;
2. Direct B/Ex-THV implantation into the native aortic valve (AV) annulus (*valve-in-ring*) – 6 cases.

### Surgical features of direct B/Ex-THV implantation into the mechanical prosthesis frame, i.e. valve-in-frame

The types of previously implanted mechanical prostheses are listed in Table 1.

**Table 1:** Direct B/Ex-THV implantation into the mechanical prosthesis frame (*valve-in-frame*) + HeartMate 3 LVAD implantation: 9 cases. The technical specifications.

Pt No	Previous surgery (Mechanical Valve Replacement)		Internal diameter, mm		MyVal sizes (mm) AV/MV positions		CPB Time (min)	Cross Clamp Time (min)	Combined surgery
	AV	MV	AV	MV	AV	MV			
1	St. Jude Masters 27		25.5		27.5		194	43	LVAD HM3
2		MEDINZH-2 27		22.1		26	210	84	LVAD HM3
3		Carbomedics Standard 29		24.2		24.5	127	41	LVAD HM3
4	St. Jude Masters 19	St. Jude Masters 31	17.8	27.2	21.5	27.5	185	86	LVAD HM3
5		St. Jude Masters 31		27.2		27.5	162	28	LVAD HM3
6		St. Jude Masters 31		27.2		27.5	107	21	LVAD HM3
7	St. Jude Masters 21	St. Jude Masters 31	19.6	27.2	20	27.5	209	51	LVAD HM3
8	MIKS 23	MIKS 29	21.0	26.6	27.5	30	146	60	LVAD HM3
9		MEDINZH-2 29		24.1		29	202	29	LVAD HM3

**Figure 1:**
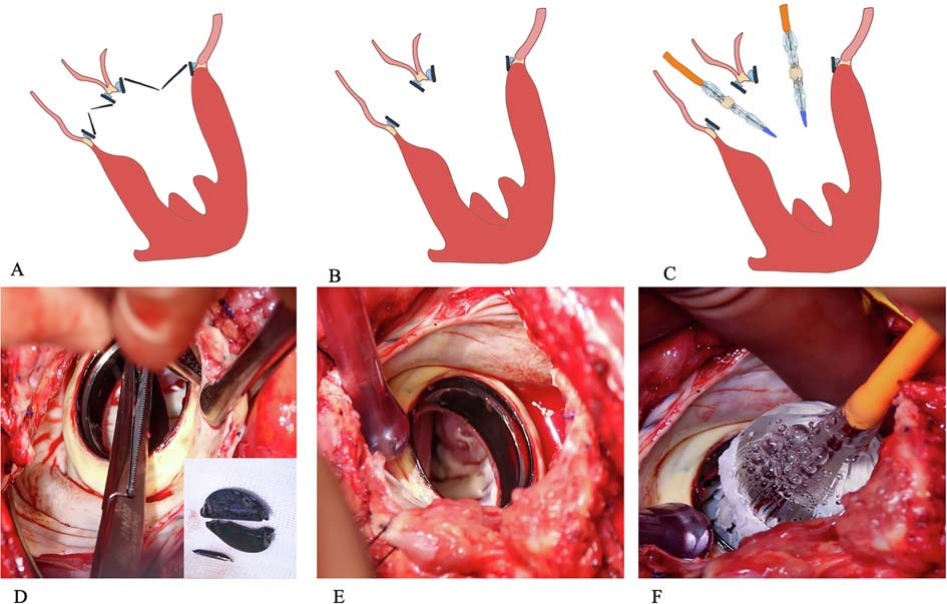
Direct B/Ex-THV implantation into the mechanical prosthesis frame (valve-in-frame) + HeartMate 3 LVAD implantation. (A. Schematic illustration of leaflet resection from the mechanical prosthesis; B. Schematic view of the mechanical frame after leaflet removal; C. Introduction of the transcatheter valve into the mechanical prosthesis frame; D. Intraoperative removal of mechanical leaflet fragments (inset: extracted components); E. View of the mechanical frame prepared for valve-in-frame implantation; F. Deployment of the transcatheter valve within the mechanical prosthesis frame).

The surgery had been performed through median sternotomy under cardiopulmonary bypass support, with aortic cross-clamp applied following the induction of cardioplegia. The mechanical prosthesis was visualized in the aortic and mitral position (Figure 1A). To dismantle the locking elements of the mechanical prosthesis, the leaflets of the prosthesis were broken and removed (Figure 1A). Next, the Navigator delivery system with a MyVal was inserted through the ascending aorta, and crimped valve was inserted into the AV and MV projection (Figure 1C).

#### Step 1. Fracture (“Cracking”) of the mechanical prosthesis valve leaflets

*Schematic* (Figure 1A) shows the intended fracture lines on the leaflet discs.

*Intraoperative view* (Figure 1D) demonstrates introduction of heavy clamp (to special leaflet-fracture forceps) through the prosthesis frame to slice and mechanically crack both discs.

*Goal*: Render the rigid leaflets non-functional and removable fragments small enough not to embolize (see inset photo of retrieved fragments).

*Key point*: Avoid damage to the surrounding prosthesis frame and metallic cage.

#### Step 2. Preparation and inspection of the prosthesis frame

*Schematic* (Figure 1B) shows the intact circular frame once the leaflets are fractured and removed.

*Intraoperative view* (Figure 1E) confirms a clean frame, with all leaflet debris cleared and the inner diameter visualized.

*Goal*: Ensure that the annular sewing ring and cage are intact and free of loose material.

*Key point*: Measure the internal diameter under direct vision (or via intra-operative TEE/CT) to select the appropriate THV size. Prior to the procedures, all patients underwent ECG-gated multislice computed tomography (MSCT) for evaluation of the aortic root and mechanical valve prosthesis frame using 3Mensio software, with parameter analysis. Additionally, the technical specifications provided by the manufacturers of the mechanical prostheses were taken into account, particularly the internal and external diameters and the height of the prosthesis (see Table 2).

**Table 2:** Direct B/Ex-THV implantation into the mechanical prosthesis frame (valve-in-frame) – 9 cases. Patients’ demographics and clinical characteristics.

No. Patients	Age (y)	Gender	Log EURO Score (%)	STS Score %	LVAD indication	INTERMACS	LV EF (%)	EDV LV, mL	ESV LV, mL	EDD LV, cm	ESD LV, cm	EF RV, %	EDD RV, cm	TAPSE, cm	Axis S/L, cm	S’RV, cm
Patient 1	36	M	67.7	22	BTT	2	11	321	287	7.5	6.9	26	3.6	0.85	0.42	8.9
Patient 2	41	M	20.42	5.2	BTT	3	21	409	321	8	7.0	28	3.5	1.6	0.43	9.1
Patient 3	50	M	35.13	11	BTT	3	16	306	255	6.5	6	33	3.0	1.65	0.52	9.0
Patient 4	40	M	32.8	14	BTT	3	17	310	256	6.6	6	30	3,6	1.6	0.44	9.0
Patient 5	45	M	25.56	12	BTT	4	23	340	261	7.1	6.7	29	2.45	1.7	0.47	9.4
Patient 6	28	M	13.03	5.5	BTT	3	25	200	149	6.4	5.1	29	2.46	1.5	0.49	8.3
Patient 7	51	M	20.47	14.8	BTT	3	8	288	263	6.9	5.8	28	4.0	1.7	0.47	8.9
Patient 8	56	F	25.52	11.2	DT	4	25	265	220	7.0	5.4	25	4.4	1.5	0.41	9.2
Patient 9	58	M	13.46	8.2	DT	4	12	360	317	8.2	7.9	29	5.8	1.6	0.52	9.0

#### Step 3. Placement of the balloon-expandable THV into the mechanical frame

*Schematic* (Figure 1C) illustrates positioning of the balloon-expandable transcatheter heart valve (B/Ex-THV) catheter within the prosthesis frame.

*Intraoperative view* (Figure 1F) captures final alignment and full balloon inflation, anchoring the THV securely into the prosthesis frame.

*Goal*: Achieve coaxial placement such that the THV skirt seals against the inner surface of the metal frame restoring a competent orifice.

*Key point*: The balloon should be inflated slowly to minimize frame deformation and reduce the risk of paravalvular leak. The valve was deployed with a balloon inflation time of approximately 5 s.

#### Step 4. Valve positioning

**Figure 2:**
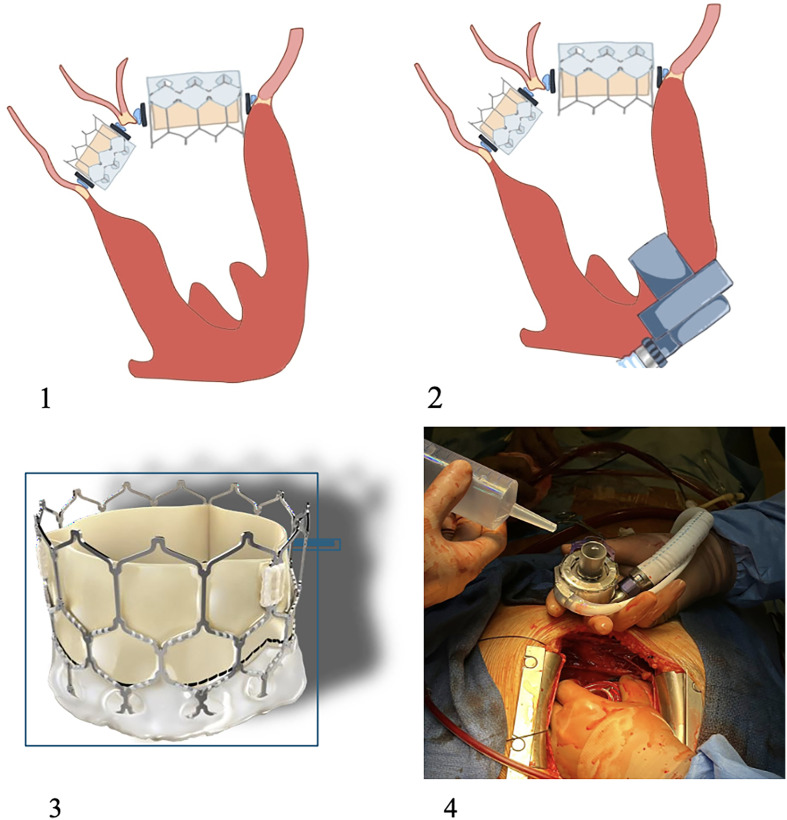
Direct B/Ex-THV implantation into the mechanical prosthesis frame (valve-in-frame) + HeartMate 3 LVAD implantation. (1. AV (MyVal 21.5) and MV (MyVal 27.5) projection; 2. Anatomical view of HeartMate 3 LVAD positioning and implanted B/Ex THV; 3. MyVal Meril; 4. Intraoperative view of HeartMate 3 LVAD.

**Figure 3:**
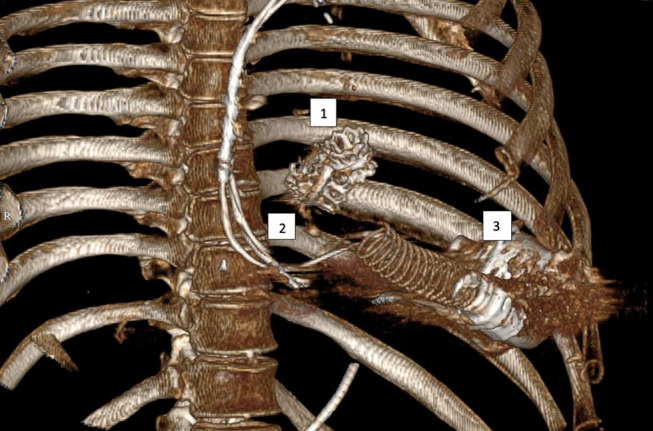
Chest CT after the procedure. 1. AV projection: valve in frame – MyVal (Meril) 21.5. 2. MV projection: valve in frame – MyVal (Meril) 27.5. 3. LVAD–Heart Mate 3.

The valve was positioned at the level of the mechanical valve ring in a ratio of 60 (AO)/40 (LVOT) (also used 70 (AO)/30 (LVOT) ratio depending on the length of aorta), the valve was deployed with a balloon inflation time of approximately 5 s (Figure 2-1). Dual nose cone on Navigator balloon ensures flawless crimping, precise positioning and predictable placement of MyVal THV (Figure 2-3). Traditionally MyVal sizes have been 20–32 mm diameters. Availability of intermediate sizes (Ø 21.5, 24.5, 27.5 mm) ensures preservation of valve geometry. Shorter height of the MyVal (Meril) THV frame prevents blockage of the coronary ostia [8]. Then, after successful positioning THV, LVAD-Heart Mate 3 (Abbott Vascular, United States) (HM 3) was implanted (Figure 2-2, 2-4).

After the surgery, there was an assessment of TEE and CT.

Prior to the procedures, all patients underwent ECG-gated multislice computed tomography (MSCT) (Figure 3) for evaluation of the aortic root and mechanical valve prosthesis frame using 3Mensio software. Additionally, the technical specifications provided by the manufacturers of the mechanical prostheses were taken into account, particularly the internal and external diameters and the height of the prosthesis (see Table 1).

### Surgical features of direct transaortic balloon expandable THV implantation into the native AV annulus (valve-in-ring)

This technique is described in more detail in the article – see [4].

**Figure 4:**
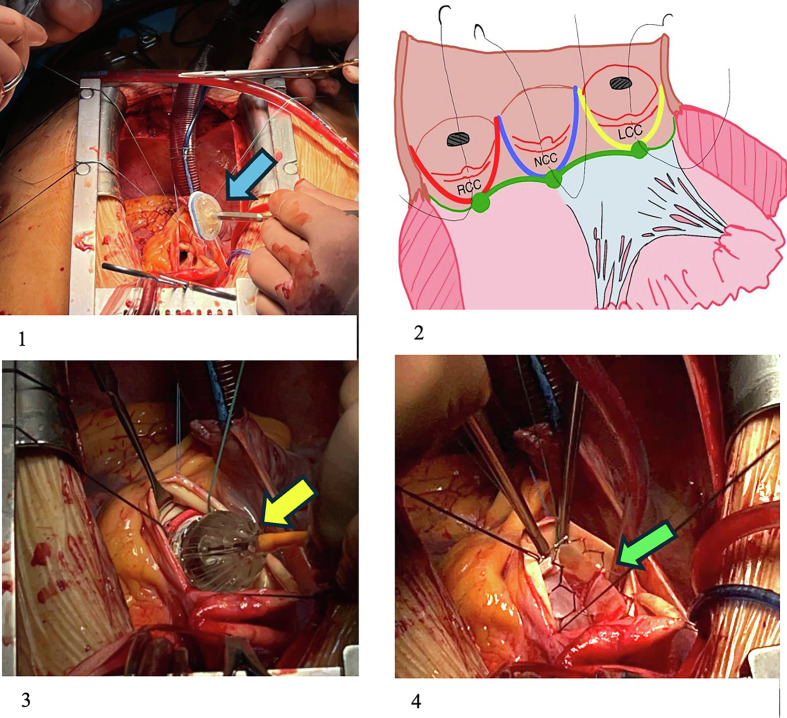
Direct B/Ex-THV implantation into the native Aortic Valve (AV) annulus **(***valve-in-ring***)** + LVAD implantation. (1. Blue arrow: flexible ring secured with three U-shaped sutures. 2. Schematic view of sutures. 3. Yellow arrow: B/Ex THV during inflation. 4. Green arrow: intraoperative view of the deployed B/Ex-THV).

The surgery also had been performed through median sternotomy under cardiopulmonary bypass support, with aortic cross-clamp applied following the induction of cardioplegia. The AV was implanted into the fibrous ring using the flexible ring (CARBOMEDICS AnnuloFlex TM32 flexible ring (Sorin Group Italia S.r.L., Italy)) with 3 U-shaped sutures Ethibond 2/0 (Johnson & Johnson Medical N.V., Belgium) (Figure 4, top left – 1) was fixed with the native annulus using the same stitches to provide a sturdy base for valve replacement [5], as illustrated in (Figure 4, top right – 2). Then, the Navigator delivery system with a MyVal cramped valve was inserted onto the projection of the flexible support ring. The valve was positioned at the level of the annulus fibrosus and deployed into the flexible ring with the appropriate aortic/LVOT proportion. Deployment was performed with a balloon inflation time of approximately 5 s. (Figure 4, bottom left – 3). Figure 4 represents the view of the MyVal – blue arrow (Figure 4, bottom right – 4). After successful THV positioning, LVAD-Heart Mate 3 (Abbott Vascular, United States) (HM 3) was implanted.

**Figure 5:**
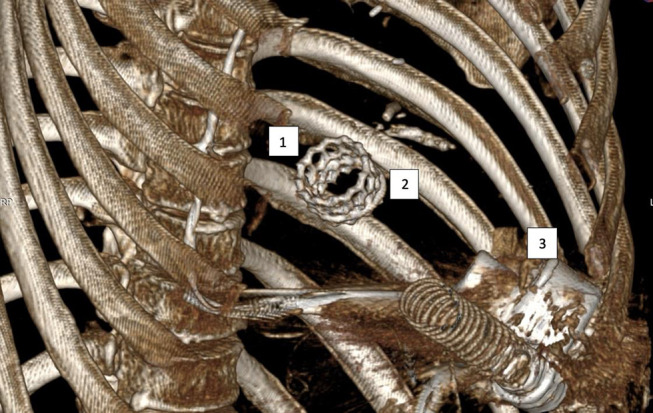
Chest CT after the procedure. 1. AV projection – valve in ring MyVal (Meril) 30.5. 2. The flexible ring Carbomedics annuloflex. 3. LVAD – Heart Mate 3.

Prior to the procedures, all patients underwent ECG-gated multiplied computed tomography (MSCT) (Figure 5) for evaluation of the aortic root using 3Mensio software (with parameter analysis) (see Table 3).

**Table 3:** Direct B/Ex-THV implantation into the native aortic valve (AV) annulus (*valve-in-ring*) + HeartMate 3 LVAD implantation: 6 cases. The technical specifications.

Pt No.	Previous surgery	Ring type and size	MyVal size (mm)	CPB time (min)	Cross clamp time (min)	Combined surgery
1		CARBOMEDICS AnnuloFlex 32	30.5	110	28	LVAD HM3
2		CARBOMEDICS Annuloflex 32	29	163	48	LVAD HM3
3	CABG	Duran AnCore 27	29	153	33	LVAD HM3
4		CARBOMEDICS Annuloflex 30	30.5	118	20	LVAD HM3
5		Duran AnCore 29	30.5	160	101	LVAD HM3
6		Duran AnCore 29	29	110	33	LVAD HM3

## Results

### Clinical insights and observations

Patients were stratified into two groups according to the anatomical site of THV implantation. The first group consisted of nine patients who received direct B/Ex-THV implantation into the frame of a previously implanted mechanical prosthesis (aortic or mitral position; valve-in-frame technique). The second group included seven patients who underwent direct B/Ex-THV implantation into the native aortic valve annulus (valve-in-ring technique). All procedures were performed concomitantly with HeartMate 3 LVAD implantation.

In the valve-in-frame group (*n* = 9) (Table 2), the mean age was 45.0 ± 9.5 years, and 66.7% (6/9) were male. The majority of patients presented in advanced stages of heart failure, with New York Heart Association (NYHA) functional class III or IV symptoms. All patients were classified as INTERMACS profiles 2 to 4, reflecting varying degrees of hemodynamic compromise. Seven patients (77.8%) underwent LVAD implantation as a bridge to transplantation (BTT), and two (22.2%) received the device as destination therapy (DT). The baseline left ventricular ejection fraction (LVEF) was severely reduced (mean 17.2 ± 5.8%). Mean left ventricular end-diastolic volume (LVEDV) was 319.3 ± 48.6 mL, and mean end-systolic volume (LVESV) was 266.7 ± 39.8 mL. Right ventricular function was variably impaired, with right ventricular ejection fraction (RVEF) ranging from 26% to 45% and tricuspid annular plane systolic excursion (TAPSE) averaging 1.45 ± 0.2 cm (Table 2).

In the valve-in-ring group (*n* = 6) (Table 4), the mean age was 60.3 ± 3.5 years, and all patients were male. All were ineligible for cardiac transplantation and underwent LVAD implantation as destination therapy. INTERMACS profiles ranged from 3 to 4, and all patients exhibited severe systolic dysfunction with a mean LVEF of 20.7 ± 4.5%. The mean LVEDV was 285.1 ± 47.9 mL, and LVESV was 243.4 ± 36.5 mL. Right ventricular function was preserved in most patients, with a mean RVEF of 41.6 ± 9.1% and TAPSE of 1.1 ± 0.2 cm (Table 4).

**Table 4:** Direct B/Ex-THV implantation into the native Aortic Valve (AV) annulus (valve-in-ring) – 6 cases. Patients’ demographics and clinical characteristics.

No. Patients	Age (y)	Gender	Log EURO Score (%)	STS Score %	LVAD indication	INTERMACS	LV EF (%)	EDV LV, mL	ESV LV, mL	EDD LV,cm	ESD LV, cm	EF RV, %	EDD RV, cm	TAPSE, cm	Axis S/L, cm	S’RV, cm
Patient 1	60	M	48.73	7.5	DT	2	29	424	299	8.7	7.7	30	3.0	1.0	0.5	11
Patient 2	61	M	20.3	8.5	DT	3	15	262	222	7.6	7.3	30	8.0	1.4	0.53	8.7
Patient 3	65	M	14.1	5.1	DT	3	18	282	220	7.9	7.0	33	5.14	1.5	0.69	9.4
Patient 4	60	M	8.94	2.3	DT	3	24	320	274	6.8	6.3	29.9	5.7	1.7	0.54	6.9
Patient 5	55	M	6.27	10	BTT	4	20	234	195	7.2	6.2	30	4.0	1.1	0.63	7.2
Patient 6	64	M	7.17	4.8	DT	3	14	290	251	7.5	6.6	28	4.9	1.4	0.52	9.0

Considering biventricular heart failure along with high risks of complications, it was preferable to reduce the time of ischemia during LVAD implantation.

Direct B/Ex-THV implantation into the Mechanical Valve (valve-in-frame) + LVAD implantation indicates a cross-clamp time (mean 49.22 min; SD 22.2) and a CPB time (mean 171.33 min; SD 35.5). Direct B/Ex-THV implantation into the native AV annulus (valve-in-ring) + LVAD implantation indicates a CPB time (mean 135.6 min; SD 23.3) and a cross-clamp time (mean 43.8 min; SD 26.8).

The ICU stay duration ranged from 1 day to 50 days. Most patients had relatively short LOS (length of stay), with 1–5 days being the most common range. Nine patients (56%) had short LOS ranging from 1 to 5 days which indicates uncomplicated post-operative course and quick recovery. Only one patient (6.25%) had a very prolonged ICU stay of 50 days. This case suggests a significant post-operative recovery issue, complications, or delayed weaning.

Of the 9 patients who underwent valve-in-frame implantation, three had procedures in both the aortic and mitral positions, one had the valve implanted in the aortic position only, and the remaining five in the mitral position only.

Table 5 summarizes the immediate and mid-term hemodynamic performance following valve-in-frame implantation in the aortic position. The mean transvalvular gradient was 5.2 ± 2.1 mmHg immediately post-implantation and remained stable at 5.0 ± 1.3 mmHg at 6–12 months of follow-up. Peak gradients were also consistent over time (12.5 ± 4.7 mmHg vs. 12.8 ± 5.2 mmHg).

**Table 5:** Immediate and mid-term hemodynamic performance after valve-in-frame implantation – AV position (*n* = 4).

Hemodynamic parameter	Immediate post-implantation	Mid-term follow-up (e.g., 6–12 months)
Mean Transvalvular Gradient (mmHg)	5.2 ± 2.1	5.0 ± 1.3
Peak Transvalvular Gradient (mmHg)	12.5 ± 4.7	12.8 ± 5.2
Effective Orifice Area (cm^2^)	1.80 ± 0.30	1.72 ± 0.35
Indexed EOA (cm^2^/m^2^)	0.90 ± 0.12	0.85 ± 0.11
Paravalvular Leak – None, *n* (%)	0 (0.0%)	0 (0.0%)
Paravalvular Leak – Mild, *n* (%)	3 (33.3%)	3 (33.3%)
Paravalvular Leak – Moderate, *n* (%)	0 (0.0%)	0 (0.0%)
Paravalvular Leak – Severe, *n* (%)	0 (0.0%)	0 (0.0%)

The effective orifice area (EOA) showed minimal decline from 1.80 ± 0.30 cm^2^ to 1.72 ± 0.35 cm^2^, and indexed EOA decreased slightly from 0.90 ± 0.12 cm^2^/m^2^ to 0.85 ± 0.11 cm^2^/m^2^. Importantly, no patients exhibited moderate or severe paravalvular leak (PVL) at any time point. Mild PVL was observed in 3 patients (33.3%) both immediately post-implantation and during mid-term follow-up, while none had complete PVL resolution.

These findings suggest preserved valve function and stable hemodynamic performance in the aortic position during mid-term follow-up after valve-in-frame implantation.

Table 6 presents the immediate and mid-term hemodynamic outcomes following valve-in-frame implantation in the mitral position. The mean transvalvular gradient remained low and stable, measured at 4.2 ± 1.7 mmHg immediately post-implantation and 4.0 ± 1.8 mmHg at 6–12 months. Peak gradients showed a modest increase over time (10.1 ± 3.7 mmHg to 12.2 ± 3.2 mmHg), yet remained within acceptable hemodynamic ranges.

**Table 6:** Immediate and mid-term hemodynamic performance after valve-in-frame implantation – MV position (*n* = 8).

Hemodynamic parameter	Immediate post-implantation	Mid-term follow-up (e.g., 6–12 months)
Mean Transvalvular Gradient (mmHg)	4.2 ± 1.7	4.0 ± 1.8
Peak Transvalvular Gradient (mmHg)	10.1 ± 3.7	12.2 ± 3.2
Effective Orifice Area (cm^2^)	2.02 ± 0.30	2.2 ± 0.35
Indexed EOA (cm^2^/m^2^)	1.0 ± 0.12	1.05 ± 0.11
Paravalvular Leak – None, *n* (%)	0 (0.0%)	0 (0.0%)

Effective orifice area (EOA) demonstrated an improvement over time, increasing from 2.02 ± 0.30 cm^2^ to 2.2 ± 0.35 cm^2^ at follow-up. Similarly, the indexed EOA increased from 1.00 ± 0.12 cm^2^/m^2^ to 1.05 ± 0.11 cm^2^/m^2^. Notably, no cases of paravalvular leak were observed at any time point, indicating excellent procedural sealing and valve seating.

These findings support the favorable hemodynamic performance and stability of valve-in-frame implantation in the mitral position during mid-term follow-up.

During the post-procedural follow-up period (*n* = 15), all patients (100%) survived to discharge. At 30 days, survival was 93.3% (14/15), with one patient progressing to right ventricular failure. Six-month survival was 86.6% (13/15), one death due to renal failure and septicemia. At both 12- and 24-month follow-up (*n* = 12), overall survival was 80% (12/15). One patient died due to gastrointestinal bleeding associated with malignancy. Stroke occurred in one patient (6.6%) within 30 days post-implantation, with no further cerebrovascular events reported during follow-up. Acute kidney injury was observed in 4 patients (26.6%) postoperatively but resolved without persistent dysfunction, with no new cases noted beyond the initial period. These findings are summarized in Table 7, which presents survival rates and major complications at defined time points up to two years.

**Table 7:** The patient outcomes and complications.

Events	Post-procedural	30-day	6-month	1 year	2-years
	FU (*n* = 16)	FU (*n* = 15)	FU (*n* = 13)	FU (*n* = 12)	FU (*n* = 12)
SURVIVAL	16 (100%)	*15 (93.75%)	^13 (81.25%)	$12 (75%)	12 (75%)
Stroke	0 (0.00)	1 (6.25)	0 (0.00)	0 (0.00)	0 (0.00)
Kidney dysfunction	4 (25.0)	0 (0.00)	0 (0.00)	0 (0.00)	0 (0.00)

Values are *n* (%).

* One Patient died due to right ventricular failure.

^ One Patient died due to renal failure and septicemia and another one died due to multiple organ failure.

$ One Patient died due to gastrointestinal bleeding associated with cancer.

FU – Follow up

During the follow-up period, no cases of hemolysis, infection, thrombosis, valve migration, paravalvular leak, myocardial infarction, or the need for a new permanent pacemaker were observed. It highlights a relatively favorable safety profile, with major mortality events primarily related to organ failure or comorbid conditions, rather than device-related issues.

One patient (Tables 1 and 2, Patient 6) who underwent valve-in-frame implantation subsequently underwent successful orthotopic heart transplantation 12 months later. The bioprosthetic valve remained well-functioning up to the time of transplantation, with no evidence of structural deterioration or significant paravalvular leak.

## Discussion

The integration of ventricular assist devices and transcatheter heart valve implantations into the clinical management of heart failure represents a paradigm shift in the treatment of advanced cardiovascular disease. Implemented together, these therapies offer patients with otherwise limited options the opportunity for improved survival, better functional status, and a significantly enhanced quality of life [8, 10, 12]. The continuous evolution of these technologies, along with advancements in patient selection and procedural techniques, will likely drive further improvements in outcomes, making them integral components of modern heart failure management [4, 12]. Repeated valve replacements combined with LVAD are complex and time-consuming procedures [8, 10]. Repeated valve replacement, particularly when combined with left ventricular assist device (LVAD) implantation, present a unique set of challenges and complexities in the management of patients with advanced heart failure (HF) [8, 10]. The need for multiple valve surgeries often emerges in patients with progressive valvular dysfunction or deteriorating heart failure symptoms, especially those with mechanical mitral or aortic valves [8]. These patients face significant risks, including prosthetic valve failure, infection, thrombosis, and bleeding complications, making repeated valve replacement both technically difficult and associated with high morbidity and mortality [11, 12]. When combined with LVAD implantation, the complexity of the procedure increases due to the need for meticulous coordination between the cardiac team, the requirement for advanced surgical techniques, and the extended time required for both valve replacement and device implantation. LVAD implantation itself is a high-risk procedure, involving careful patient selection, surgical skill, and post-operative management [1]. Combining this with valve replacement, particularly in patients with previous valve prostheses, often requires addressing additional anatomical challenges, such as altered valve positions, scar tissue, and aortic or mitral root abnormalities [13, 14]. The length and invasiveness of such procedures pose a significant burden on patients, leading to longer recovery and higher likelihoods of complications [13, 14]. Cardiopulmonary bypass (CPB) time, which is a critical factor in these surgeries, can be prolonged due to the complexities involved in both replacing the valve and ensuring proper LVAD function [12, 13]. Furthermore, these procedures often involve multiple teams of specialists, including cardiologists, cardiothoracic surgeons, and anesthesiologists, in order to manage the wide range of potential complications [4].

Given the inherent risks of repeated valve replacements and LVAD implantation, novel strategies are being explored to minimize complications and improve patient outcomes. One such approach involves using advanced devices like the B/Ex valve, which offers opportunity for direct valve implantation that significantly reduces ischemic time and avoids extended surgical period [4, 7].

In patients with end-stage heart failure (HF), the combined need for durable mechanical circulatory support and concomitant valvular intervention poses significant clinical and technical challenges. This study presents a novel approach – direct implantation of a balloon-expandable transcatheter heart valve (B/Ex-THV) either into the frame of a previously implanted mechanical prosthesis or directly into the native aortic valve annulus – performed simultaneously with HeartMate 3 left ventricular assist device (LVAD) implantation [4]. The procedure was applied in a cohort of high-risk patients with extensive surgical histories and significant comorbidities, in accordance with the International Society for Heart and Lung Transplantation (ISHLT) guidelines [3, 6]. The ISHLT 2023 consensus discourages the use of mechanical aortic valves in LVAD recipients, as their continuous flow reduces leaflet mobility and increases thrombogenic risk [6, 12]. Mechanical mitral valves pose similar risks, potentially exacerbating inflow obstruction or high transvalvular gradients due to the altered flow dynamics associated with LVADs [3]. Our findings support these recommendations by demonstrating that transcatheter valve implantation using a B/Ex-THV in place of mechanical prostheses or native valve replacement is not only feasible but also clinically advantageous in this population.

In this context, direct B/Ex-THV placement was selected as a means to address the dual challenge of valve pathology and advanced heart failure, while avoiding the risks of conventional reoperation. The transcatheter approach allows for: Reduction in total CPB and cross-clamp times; Lower incidence of bleeding, thrombosis, and right heart dysfunction; A simplified and reproducible surgical strategy for high-risk redo patients.

Two B/Ex-THV implantation strategies were employed: (1) valve anchoring within a pre-existing mechanical prosthesis frame – valve-in-frame (*n* = 9), and (2) implantation into the native aortic annulus – valve in ring (*n* = 6). This dual approach allowed tailored therapy for each anatomical and clinical scenario while utilizing a single surgical field. The procedural strategy significantly minimized cardiopulmonary bypass (CPB) and myocardial ischemic time – factors known to impact right ventricular function and overall surgical outcomes in LVAD recipients. By reducing ischemia-reperfusion injury and limiting total surgery time, this method has the potential to reduce right ventricular failure and early postoperative morbidity, which remain among the leading causes of death in this population.

Hemodynamically, the use of B/Ex-THV demonstrated favorable outcomes. The transcatheter bioprostheses preserved forward flow in low-pressure environments without causing leaflet immobility or thrombus formation. Importantly, the strategy avoids the lifelong anticoagulation associated with mechanical valves – an especially important consideration in patients with bleeding risks or frailty. In our cohort, paravalvular leak was absent or minimal, and early valve performance metrics, including transvalvular gradients and effective orifice area, were within expected ranges.

Patient selection for this approach followed ISHLT and current guideline-based criteria for advanced HF and structural heart interventions. All included patients met criteria for LVAD implantation either as bridge to transplantation (BTT) or destination therapy (DT) and had significant valvular pathology or redo surgery risk. The presence of multiple prior sternotomies, a hostile mediastinum, and/or high STS or EuroSCORE II risk scores justified a minimally invasive, hybrid strategy [14]. Notably, 10 of the 16 patients had a history of prior open-heart surgery, reinforcing the need for innovation in this high-risk group.

By integrating valve therapy into the same operative session as LVAD implantation, we not only simplified the procedural workflow but also potentially improved long-term outcomes. This approach offers particular utility in redo patients with mechanical prostheses who are poor candidates for complex surgical re-replacement. Furthermore, the use of imaging-based frame sizing and preoperative planning proved essential for procedural success, underlining the importance of advanced CT-based techniques in structural heart therapy.

This case series demonstrate that direct B/Ex-THV implantation, in conjunction with LVAD placement, is a viable alternative to traditional surgical techniques in selected high-risk HF patients. The strategy aligns with evolving guidelines, optimizes perioperative conditions, and preserves valve function in the unique hemodynamic environment of continuous-flow LVAD support. Further multicenter studies and long-term follow-up will be necessary to confirm durability, assess survival benefit, and refine patient selection criteria.

By embedding this method into standard LVAD implantation workflows, we not only improve procedural efficiency but also align clinical practice with evolving international standards. This combined strategy represents a viable path forward for optimizing outcomes in a complex and growing patient population.

Previous reports have described only isolated cases of MyVal implantation in LVAD recipients, including the first successful TAVI with MyVal in a HeartMate II patient with de novo aortic regurgitation and, more recently, the first case in a HeartMate III patient treated with the same device [15]. These pioneering experiences highlighted the feasibility of expanding B/Ex-THV therapy to patients supported with durable LVADs.

This is one of the first reported series describing the use of B/Ex-THV in the mitral position within the frame of a pre-existing mechanical prosthesis in patients undergoing LVAD implantation. This approach offers a novel solution for high-risk redo patients, where removal of the prosthetic frame would significantly prolong operative and cardiopulmonary bypass time. These findings expand the current evidence base and underscore the potential role of THV therapy in complex redo scenarios in LVAD patients.

## Conclusion

The selected cohort represents a high-risk population requiring LVAD therapy with concurrent structural valve disease. Given the limitations of mechanical prostheses in LVAD patients (e.g., thrombosis risk, hemodynamic mismatch, and anticoagulation concerns), B/Ex-THV implantation was chosen as the preferred strategy for redo cases. This approach minimizes procedural complexity, reduces ischemic time, and improves post-LVAD hemodynamic stability. The described direct B/Ex-THV implantation into a mechanical valve prosthesis followed by LVAD implantation represents a feasible and effective surgical approach in redo cardiac surgery patients with high perioperative risk. The combined use of balloon-expandable THV and LVAD technology provides a minimally invasive, durable solution, significantly reducing ischemic time and postoperative complications while adhering to ISHLT guidelines for LVAD patient management.

The use of B/Ex-THV implantation in LVAD patients with previous valve surgery represents a novel, ISHLT aligned strategy that optimizes surgical outcomes, hemodynamic stability, and patient survival. A key advantage of this strategy is its strict adherence to ISHLT recommendations, which reinforces both its novelty and clinical relevance. This approach addresses critical limitations associated with mechanical prostheses in LVAD recipients, ensuring a safer, more effective intervention for redo patients with complex cardiovascular histories. Direct balloon expandable transcatheter heart valve implantation markedly reduces the duration of the procedure, which surely is going to have a beneficial effect in postoperative period of the patient and good outcomes in short and long-term support. These approaches are used to improve prognosis and quality of life in patients with end-stage heart failure.

## Data Availability

The original contributions presented in the study are included in BR28712377). The article/supplementary material, further inquiries can be directed to the corresponding author.

## References

[1] Goldstein DJ, Naka Y, Horstmanshof D, et al. Association of clinical outcomes with left ventricular assist device use by bridge to transplant or destination therapy intent: The multicenter study of MagLev technology in patients undergoing mechanical circulatory support therapy with HeartMate 3 (MOMENTUM 3) randomized clinical trial. JAMA Cardiol. 2020;5(4):411–419. 10.1001/jamacardio.2019.5323. 31939996 PMC6990746

[2] Felker GM, Rogers JG. Same bridge, new destinations rethinking paradigms for mechanical cardiac support in heart failure. J Am Coll Cardiol. 2006;47(5):930–932. 10.1016/j.jacc.2005.09.070. 16516073

[3] Seth A, Kumar V, Singh VP, Kumar D, Varma P, Rastogi V. Myval: A novel transcatheter heart valve for the treatment of severe aortic stenosis. Interv Cardiol. 2023;18:e12. 10.15420/icr.2020.32. 37398875 PMC10311401

[4] The 2023 ISHLT guidelines for mechanical circulatory support: A 10-year update: J Heart Lung Transplantation 2023;42(7):E1–222. 10.1016/j.healun.2022.12.004 37245143

[5] Pya Y, Mussayev A, Novikova S, et al. Case report: A novel surgical technique for rapid valve-in-ring implantation into the native aortic annulus during left ventricular assist device implantation. Front Cardiovasc Med. 2023;10:1091420. 10.3389/fcvm.2023.1091420. 37089890 PMC10117784

[6] Kilic T, Coskun S, Mirzamidinov D, Yilmaz I, Yavuz S, Sahin T. Myval transcatheter heart valve: The future of transcatheter valve replacement and significance in current timeline. J Clin Med. 2024;13(22):6857. 10.3390/jcm13226857. 39598000 PMC11594825

[7] Kiefer P, Hoyer A, Borger MA, Garbade J. Direct transaortic transcather valve-in-valve implantation into a mechanical aortic valve prosthesis during left ventricular assist device implantation: description of a surgical technique. Interact Cardiovasc Thorac Surg. 2022;34(2):329–330. 10.1093/icvts/ivab253. 34697643 PMC8782224

[8] Seth A, Kumar V, Singh VP, Kumar D, Varma P, Rastogi V. Myval: A novel transcatheter heart valve for the treatment of severe aortic stenosis. Interv Cardiol. 2023;18:e12. 10.15420/icr.2020.32. 37398875 PMC10311401

[9] Zaidi SH, Minhas AMK, Sagheer S, et al. Clinical outcomes of transcatheter aortic valve replacement (TAVR) vs. surgical aortic valve replacement (SAVR) in patients with durable left ventricular assist device (LVAD). Curr Probl Cardiol. 2022;47(10):101313. 10.1016/j.cpcardiol.2022.101313. 35817155

[10] Sattar Y, Song D, Almas T, et al. Cardiovascular outcomes and trends of transcatheter vs. surgical aortic valve replacement among octogenarians with heart failure: A propensity matched national cohort analysis. Int J Cardiol Heart Vasc. 2022;42:101119. 10.1016/j.ijcha.2022.101119. 36161232 PMC9489740

[11] Al Saadi T, Andrade A, Chickerillo K, et al. A case series of patients with left ventricular assist devices and concomitant mechanical heart valves. Artif Organs. 2020;44(10):1050–1054. 10.1111/aor.13702. 32279355

[12] Kadakia S, Moore R, Ambur V, Toyoda Y. Current status of the implantable LVAD. Gen Thorac Cardiovasc Surg. 2016;64(9):501–508. 10.1007/s11748-016-0671-y. 27270581

[13] Ayers BC, Wood K, McNitt S, et al. Association of previous cardiac surgery with outcomes in left ventricular assist device patients, Interactive CardioVascular and Thoracic Surgery, 2020;31(1):1–8. 10.1093/icvts/ivaa055. 32248242

[14] Roselli EE, Pettersson GB, Blackstone EH. Adverse events during reoperative cardiac surgery: frequency, characterization, and rescue. J Thorac Cardiovasc Surg. 2008;135(2):316–323. 10.1016/j.jtcvs.2007.08.060. 18242260

[15] Hamieh M, Nassereddine Z, Moussa M, Al Ali F, Dbouk M, Saab M. Transcatheter aortic valve implantation for aortic regurgitation in HeartMate II supported patient using Myval THV: A case report. Oxf Med Case Reports. 2023;2023(10):omad086. 10.1093/omcr/omad086. 37881260 PMC10597617

